# NSUN5 as a Prognostic Biomarker Correlates with Malignant Phenotype and Therapeutic Target in Glioma

**DOI:** 10.1002/brb3.71211

**Published:** 2026-01-19

**Authors:** Ye Wenhao, Wu Huan, Zou Xiaoyun, Yang Yuanyuan, Bi Junlei, Liu Changqing, Zhao Mengyi, Zhang Yuyuan, Lu Jin, Wen Hebao, Ma Caiyun

**Affiliations:** ^1^ Anhui Engineering Research Center for Neural Regeneration Technology and Medical New Materials Bengbu Medical University Bengbu China; ^2^ West China School of Basic Medical Sciences and Forensic Medicine Sichuan University Chengdu China; ^3^ School of Humanities and Health Bengbu Medical University Bengbu China; ^4^ School of Life Science Bengbu Medical University Bengbu China

**Keywords:** glioma, NSUN5, prognostic biomarker, therapeutic target

## Abstract

**Background:**

NSUN5 is a conserved RNA methyltransferase whose oncogenic role has been demonstrated in various cancers. However, its function and prognostic value in gliomas remain unclear.

**Methods:**

In this study, we systematically analyzed the expression and functional associations of NSUN5 in glioma using data from The Cancer Genome Atlas (TCGA) and the Chinese Glioma Genome Atlas (CGGA) databases. A total of 117 machine learning algorithm combinations were employed to construct and validate a prognostic model for glioma patients. In addition, in vitro experiments were performed to further validate the expression and biological functions of NSUN5.

**Results:**

NSUN5 expression is significantly upregulated in glioma and is positively associated with tumor malignancy and poor prognosis. Immune infiltration analysis revealed a marked increase in M2 macrophages in the NSUN5 high‐expression group, and NSUN5 levels were positively correlated with the expression of multiple inhibitory immune checkpoints. In addition, drug sensitivity analysis and molecular docking suggested that NSUN5 may influence the response to Olaparib. Finally, based on NSUN5‐associated genes, we constructed 117 machine learning models and identified the optimal prognostic model, STRICOM, which demonstrated robust predictive performance for patient survival.

**Conclusion:**

High NSUN5 expression is closely associated with poor prognosis in glioma patients, highlighting its potential as a prognostic biomarker and therapeutic target.

## Graphical abstract

This study systematically analyzed the expression levels, clinical features, prognostic value, immune‐related roles, drug sensitivity, and biological functions of NSUN5 in glioma based on the CGGA and TCGA databases. Finally, a machine learning‐based prognostic model was constructed, and the function of NSUN5 was validated through in vitro experiments.

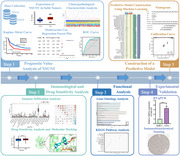

## 1 Introduction

Glioblastoma (GBM) is highly invasive and associated with a significant risk of recurrence, representing the most common primary brain tumor, accounting for 80% of all primary malignant brain tumors in adults (Finch et al. 2021). In 2021, the World Health Organization (WHO) reclassified Grades I and II gliomas as low‐grade and Grades III and IV gliomas as high‐grade (Louis et al. 2021). Low‐grade gliomas exhibit lower malignancy, leading to more favorable prognoses for patients. In contrast, high‐grade gliomas constitute the majority of gliomas (over 70%) and are highly malignant, prone to recurrence, and associated with a poor overall prognosis. Statistical data show that the median survival for Grade III glioma patients after treatment ranges from 3 to 5 years, while for Grade IV glioma patients, even with aggressive treatment, the median survival time is approximately 15 months (Fathi Kazerooni et al. 2021; Louis et al. 2021; Wagner‐Ballon et al. 2025). Conventional therapies, including surgical resection, radiotherapy, and temozolomide (TMZ) chemotherapy, have yielded unsatisfactory outcomes in GBM patients (Fasano et al. 2024). Therefore, there is an urgent need to identify novel therapeutic strategies for glioma. In recent years, molecular characteristics have been increasingly utilized for glioma classification and associated with tumor biology and clinical prognosis. Mutations in isocitrate dehydrogenase (IDH) and codeletions of chromosomes 1p/19q are considered prognostic factors that significantly impact patient survival (Chen et al. 2017; Yan et al. 2009). Therefore, identifying reliable and distinctive biomarkers for glioma diagnosis and treatment is essential for improving survival rates and prognosis in glioma patients.

NSUN5 is an RNA methyltransferase involved in various biological processes. Studies have shown NSUN5 regulates ferritin heavy chain 1 in gastric cancer, inhibits ferroptosis, and promotes tumor cell proliferation (Su et al. 2023). In clear cell renal cell carcinoma, NSUN5 overexpression enhances cancer cell invasion, proliferation, and migration while inhibiting apoptosis via suppression of the p53 signaling pathway (Li et al. 2023). NSUN5 functions as a tumor promoter in colorectal cancer by regulating the cell cycle (Jiang et al. 2020). In liver cancer, NSUN5 expression is elevated in patient tissues relative to normal liver tissues, and gene knockout notably inhibits liver cancer cell proliferation (Gu et al. 2024). NSUN5 and ALYREF are upregulated during the metastatic stages of head and neck squamous cell carcinoma, suggesting a role in metastasis (Zhu et al. 2023). Increasing evidence suggests that NSUN5 is aberrantly expressed in both solid tumors and neurological diseases and is closely linked to patient survival. However, the role and underlying mechanisms of NSUN5 in gliomas are still unclear.

**FIGURE 1:**
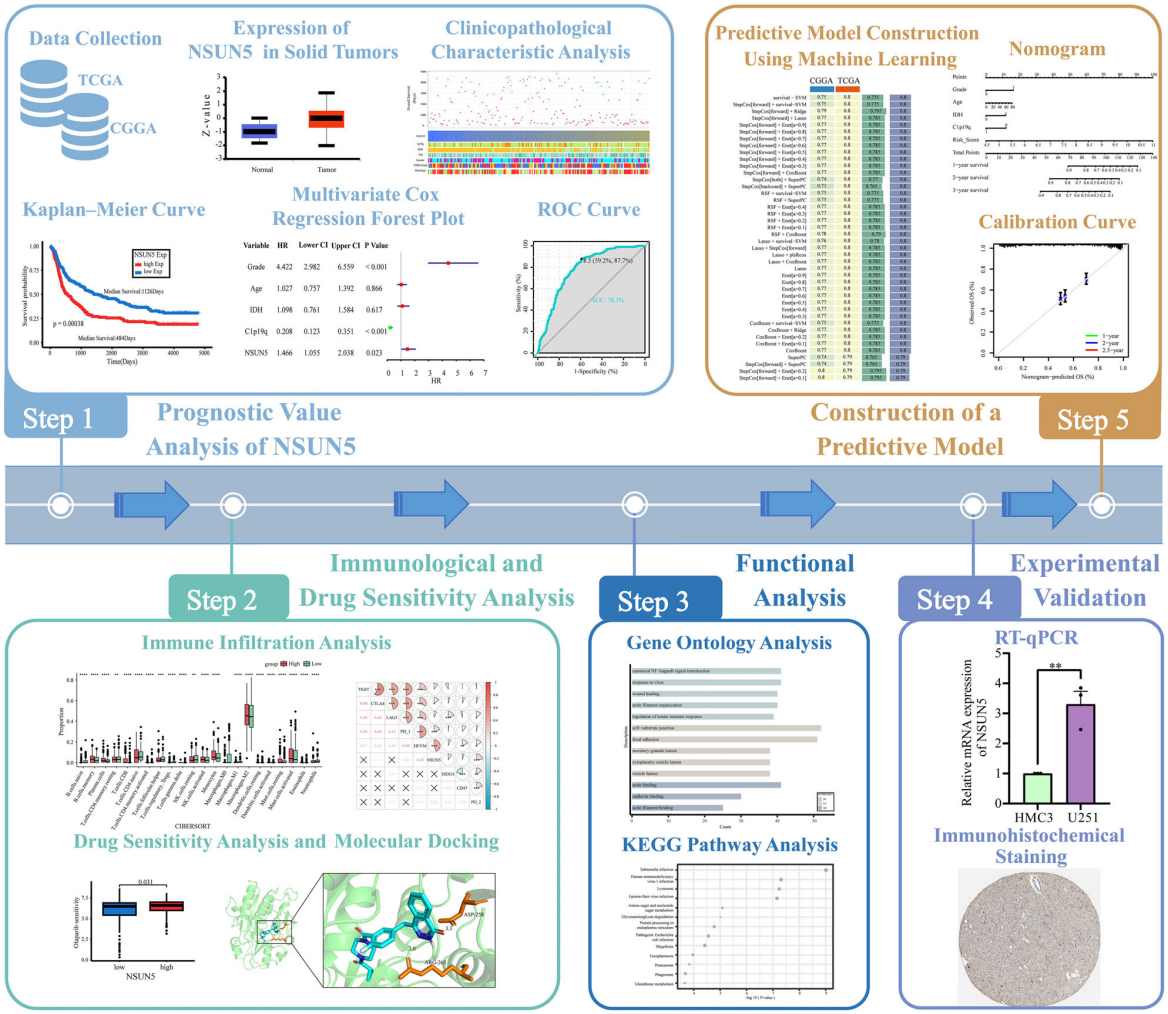
Overall workflow of the study.

This study thoroughly examined the expression levels, clinical features, biological functions, and prognostic value of NSUN5 in gliomas, utilizing a training cohort of 325 patients from the Chinese Glioma Genome Atlas (CGGA) and a validation cohort of 702 patients from The Cancer Genome Atlas (TCGA). The study identified potential mechanisms through which NSUN5 contributes to poor outcomes in glioma patients, offering insights for future targeted therapies. The workflow of this study is shown in Figure 1.

## 2 Materials and Methods

### 2.1 Data Collection and Preprocessing

First, we retrieved raw data from official sources, including clinical and RNA sequencing data of glioma patients from the CGGA (DataSet ID: mRNAseq_325, http://www.cgga.org.cn/) and TCGA databases (DataSet ID: TCGA RNAseq, https://www.cancer.gov/ccg/research/genome‐sequencing/tcga). We also gathered expression data from normal human brain tissues (*n* = 20) as controls (DataSet ID: mRNA sequencing (non‐glioma control, http://www.cgga.org.cn/). The summarized data are presented in Table 1.

**TABLE 1:** Clinical information of patients.

CGGA Database	No. of patients (*n* = 325)		TCGA database	No. of patients (*n* = 702)	
Age			Age		
>40		195	>40		385
<40		130	<40		224
NA		0	NA		93
Gender			Gender		
Male		122	Male		394
Female		203	Female		255
NA		0	NA		93
WHO grade			WHO grade		
II		103	II		216
III		79	III		241
IV		139	IV		152
NA		4	NA		93
IDH mutation			IDH mutation		
Wildtype		149	Wildtype		234
Mutant		175	Mutant		428
NA		1	NA		40
1p/19q codeletion			1p/19q codeletion		
Non‐codel		250	Non‐codel		495
Codel		67	Codel		169
NA		8	NA		38

Abbreviation: No. of patients, number of patients.

### 2.2 Differential Expression and Mutation Analysis of NSUN5 in Solid Tumors

To investigate the differential expression of NSUN5 across various solid cancers, we utilized the Proteomics module of the UALCAN database (http://ualcan.path.uab.edu/), with parameters set as Gene Name: NSUN5 and CPTAC dataset (https://cptac‐data‐portal.georgetown.edu/cptacPublic): GBM Multiforme. We analyzed NSUN5 expression across pan‐cancers and in glioma patients stratified by age, sex, and body mass index (BMI). Furthermore, we selected the GBM Multiforme (TCGA, PanCancer Atlas) dataset (n = 562) from the cBioPortal database (https://www.cbioportal.org/). Protein levels were quantified using *Z*‐scores obtained from reverse‐phase protein array (RPPA) technology, and mutation analysis of NSUN5 was performed.

### 2.3 Prognostic Analysis of NSUN5

We first downloaded gene sets for four transcriptomic subtypes of glioma from the GSEA platform (http://software.broadinstitute.org/gsea/). Using the “GSVA” R package, we evaluated NSUN5 expression across different transcriptomic subtypes by integrating patient expression data. The specificity of the results was subsequently validated using receiver operating characteristic (ROC) curve analysis. Next, Kaplan–Meier survival curves were generated using the “survminer” and “survival” packages in R to compare overall survival (OS) between patients with high and low NSUN5 expression. Univariate Cox proportional hazards regression analysis was then performed using IBM SPSS Statistics 26 to identify significant prognostic variables (*p* < 0.05). Significant variables were further subjected to multivariate Cox regression analysis using the “forestplot” package in R.

### 2.4 Immune Infiltration Analysis

CIBERSORT‐based immune infiltration data were obtained via the TIMER platform to compare differences in the infiltration levels of 22 immune cell types between NSUN5 high‐ and low‐expression groups and to assess their correlations. Furthermore, Pearson correlation analysis was conducted to evaluate the associations between NSUN5 expression and multiple commonly studied immune checkpoint molecules.

### 2.5 Drug Sensitivity Analysis

The Gene Set Cancer Analysis (GSCA) database was utilized to identify drugs correlated with NSUN5 gene expression. Subsequently, GDSC2 expression and drug response data were retrieved from the Genomics of Drug Sensitivity in Cancer (GDSC) database. The oncoPredict package performed drug sensitivity analysis on CGGA and TCGA patient cohorts.

### 2.6 Molecular Docking

The structure of the NSUN5 protein was obtained from the UniProt database (https://www.uniprot.org/), and the small‐molecule structures of the candidate drugs were retrieved from the PubChem database (https://pubchem.ncbi.nlm.nih.gov/). Redundant water molecules and heteroatoms were removed using PyMOL software. The processed protein and ligand structures were submitted to the CB‐Dock2 platform (https://cadd.labshare.cn/cb‐dock2/php/index.php) for molecular docking analysis. The docking results were subsequently visualized using PyMOL.

### 2.7 WGCAN Functional Enrichment Analysis

We conducted Weighted Gene Co‐expression Network Analysis (WGCNA) on glioma transcriptomic data to identify the most relevant gene sets. Furthermore, Pearson correlation analysis was performed on the most relevant gene sets with thresholds of *p* < 0.05 and correlation coefficient (cor) > 0.3 to identify genes associated with NSUN5, which were subsequently analyzed using Gene Ontology (GO) and KEGG pathway analyses.

### 2.8 Development of an Optimal Prognostic Model Using 117 Machine Learning Combinations

A recently developed machine learning framework, Mime, was employed to construct optimal prognostic models based on input variables and cohort data. This framework integrates ten classical machine learning algorithms and four feature selection methods (Lasso, StepCox, CoxBoost, and RSF), resulting in 117 parameter combinations. Detailed algorithm parameters are provided in Table S1. All 117 combinations were included in the computational framework and subjected to K‐fold cross‐validation on the training set. In the validation set, the model with the highest average concordance index (C‐index) was considered optimal, balancing predictive accuracy with a low risk of overfitting. Based on co‐expressed genes, we used the Mime package to build the optimal model using the CGGA training cohort and TCGA validation cohort. Patients were stratified into high‐ and low‐risk groups using the median of the risk scores calculated by the machine learning model. The optimal cut‐off value was determined using the survplot function in R, and Kaplan–Meier survival curves were generated accordingly.

### 2.9 Performance Evaluation of the Optimal Model

Time‐dependent ROC curve analysis was performed using the Mime package to calculate the 1‐, 3‐, and 5‐year area under the curve (AUC) values for the top 15 ranked models across all cohorts, in order to evaluate the predictive performance of the optimal model among the 117 combinations. Furthermore, the Mime package incorporates 95 previously published glioma prognostic models (including both LGG and GBM). We compared the hazard ratio (HR), C‐index, and the AUC values at 1, 3, and 5 years of our optimal model with those of the established models to assess its relative predictive power. The distinguishing features and their corresponding coefficients are provided in the Mime R package and are detailed in Table S2. To further evaluate the prognostic value of the optimal model, we performed a univariate Cox regression meta‐analysis based on its risk score, followed by multivariate Cox regression incorporating several known risk factors.

### 2.10 Construction and Validation of the Nomogram

Based on WHO grade, age, 1p/19q codeletion status, IDH mutation status, and risk score from the CGGA training cohort and TCGA validation cohort, a nomogram was constructed using the rms package to predict 1‐, 2‐, and 3‐year OS probabilities for glioma patients. The calibration curves were subsequently used to evaluate the concordance and accuracy of the nomogram predictions.

### 2.11 RNA Isolation and Reverse Transcription Quantitative PCR (RT‐qPCR)

RNA was extracted from cells using TRIzol reagent (Invitrogen) following the manufacturer's instructions. cDNA was synthesized using SuperScript II reverse transcriptase (Invitrogen) in the presence of an RNase inhibitor. The reaction was carried out using an Eppendorf Mastercycler eprealplex system. PCR primer sequences are listed in Table S3.

### 2.12 Statistical Analysis and Visualization

Statistical analyses and graph generation in this study were primarily performed using R software (version 4.3.2, Windows platform), IBM SPSS Statistics (version 26, Windows platform), GraphPad Prism (version 9, Windows), PyMOL (version 3.1.4, Windows), and Adobe Illustrator 2024 (Windows). Various R packages, including ggplot2, pheatmap, readxl, forestplot, GSVA, GSEABase, tibble, ggpubr, survminer, survival, Matrix, foreach, glmnet, circlize, corrgram, dplyr, Seurat, patchwork, and pROC, were used for data visualization and analysis. Additionally, IBM SPSS Statistics was employed for univariate and multivariate Cox regression analyses. Pearson correlation analysis was used to evaluate the Relationship between two data sets. Unpaired *t*‐tests were used to assess the significance of differences between the two groups. In comparison, one‐way analysis of variance (ANOVA) was employed to evaluate differences among more than two groups. A *p* value of < 0.05 was considered statistically significant (ns, *p* > 0.05; ^*^ *p* < 0.05; ^**^ *p* < 0.01).

## 3 Results

### 3.1 Expression of NSUN5 in Solid Tumors

**FIGURE 2:**
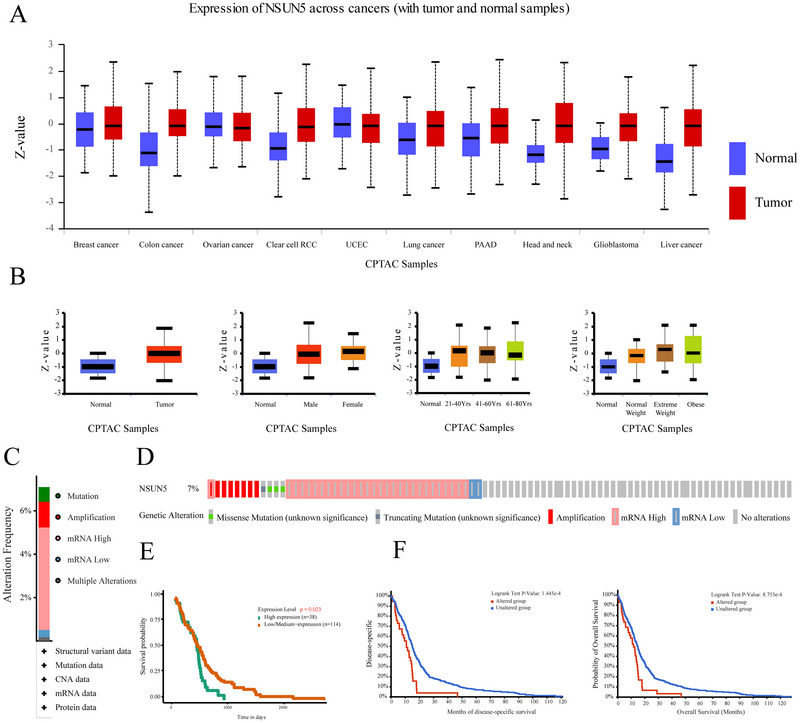
Expression of NSUN5 in solid cancer. (A) Expression of NSUN5 in solid tumors from the CPTAC database. (B) Expression of NSUN5 in glioma from the CPTAC database. (C) Proportion of NSUN5 mutations in tissue samples. (D) Mutation frequency of NSUN5 in glioma. (E) Kaplan–Meier survival curves stratified by NSUN5 expression in glioma patients. (F) Relationship between NSUN5 mutations and survival prognosis in glioma patients.

First, we analyzed the CPTAC database and found that NSUN5 expression was significantly increased in colorectal cancer, clear cell renal carcinoma, lung cancer, pancreatic cancer, head and neck cancer, gliomas, and liver cancer (Figure 2A). In glioma, we observed that NSUN5 expression was significantly elevated across patients of different sexes, ages, and body weights (Figure 2B). Kaplan–Meier survival analysis revealed that patients with high NSUN5 expression exhibited significantly reduced OS (Figure 2E). Using the cBioPortal website, we conducted a mutation analysis of NSUN5. We found that its mutation rate in gliomas reached 7%, with five mutation types: missense mutations, truncating mutations, amplification, mRNA high expression, and mRNA low expression (Figure 2C,D). These mutations influenced disease specificity and OS probability in glioma patients (Figure 2F).

### 3.2 High Expression of NSUN5 in Tumors with Higher Malignancy

**FIGURE 3:**
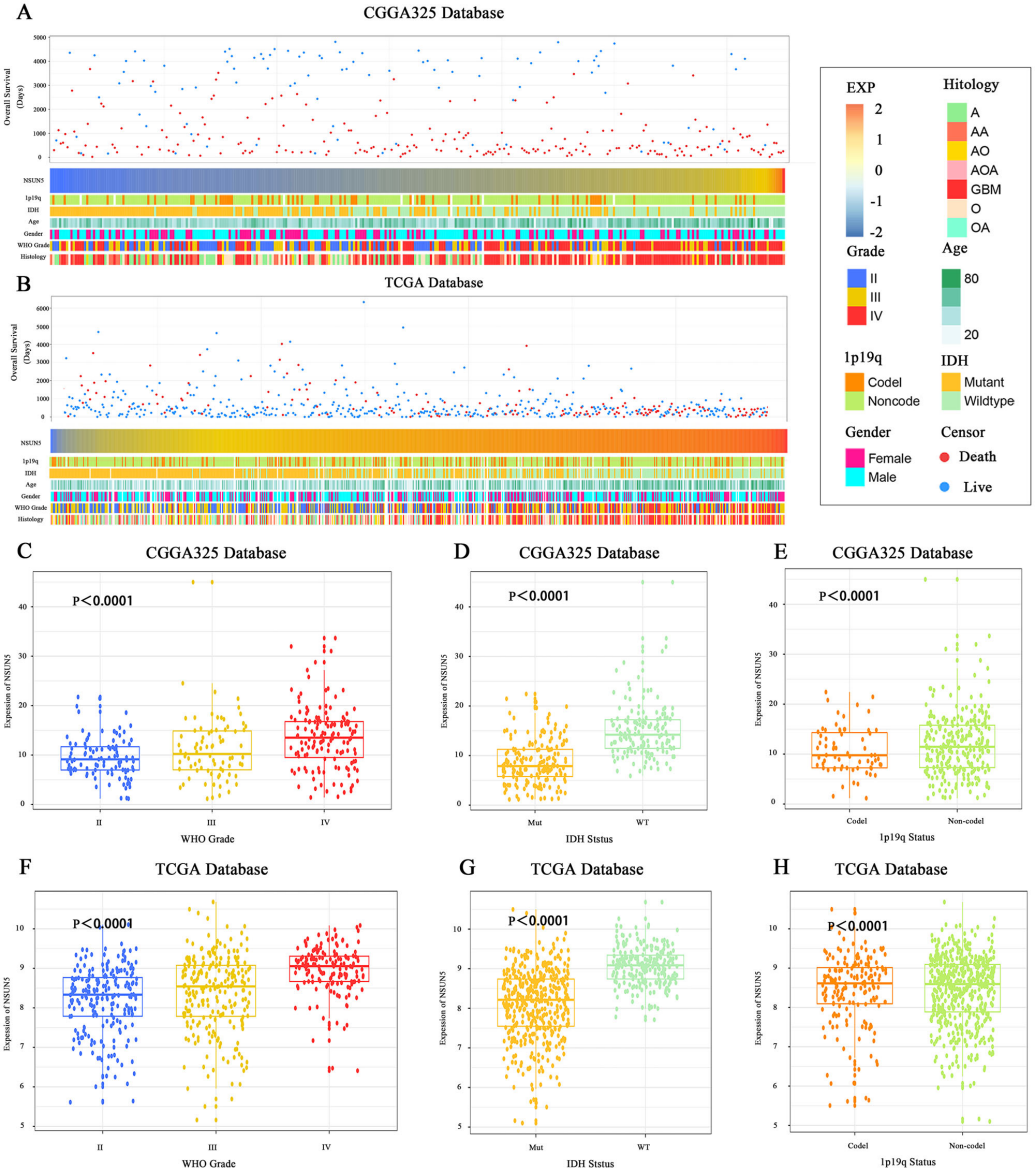
Association of NSUN5 with clinical and pathological features of glioma. (A), (B) Status of NSUN5 expression and association with clinical and pathological features of glioma in the CGGA and TCGA databases. (C), (F) NSUN5 expression is significantly elevated in high‐grade glioma (one‐way ANOVA *p* < 0.001). (D), (G) NSUN5 expression significantly increases in IDH wild‐type gliomas (unpaired *t*‐test, *p* < 0.001). (E), (H) NSUN5 expression is significantly elevated in 1p/19q non‐co‐deleted gliomas (unpaired *t*‐test, *p* < 0.001).

Patients with different levels of NSUN5 expression exhibit distinct clinical and pathological features. As NSUN5 expression increases, the distribution of 1p/19q codeletion status, IDH mutation status, WHO grade, and histological diagnosis in the CGGA and TCGA datasets is not random (Figure 3A,B). In the CGGA database, NSUN5 expression was higher in patients with high‐grade gliomas (Figure 3C), IDH‐wildtype gliomas (Figure 3D), and those without 1p/19q codeletion (Figure 3E). Validation using the TCGA database showed that the results are highly consistent with those from the CGGA training set (Figure 3F,H). Moreover, we found that OS in glioma patients significantly decreases as NSUN5 expression increases (Figure 3A,B).

### 3.3 NSUN5 as an Independent Prognostic Marker for Overall Survival in Glioma Patients

**FIGURE 4:**
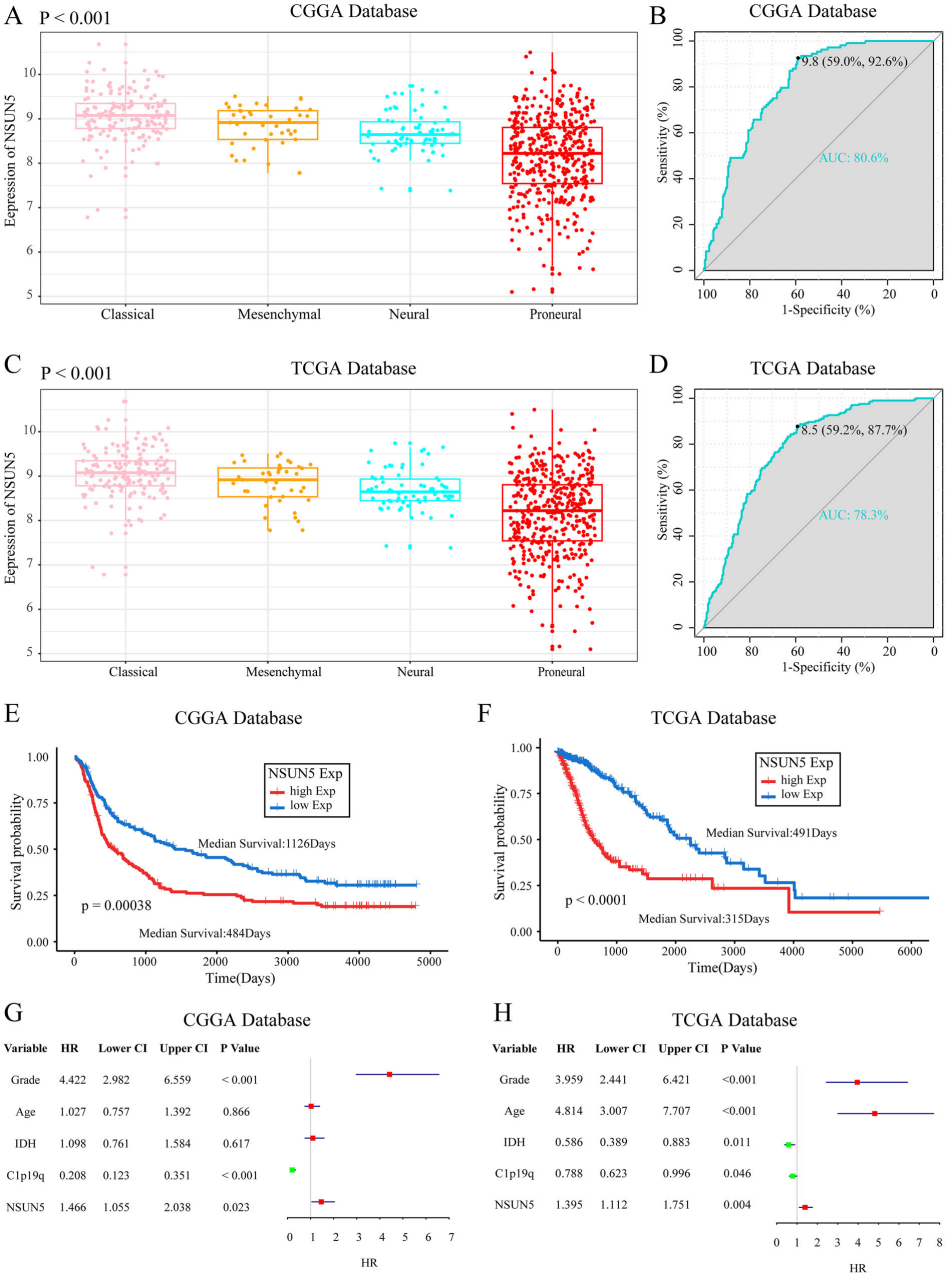
Prognostic value of NSUN5. (A), (C) Expression of NSUN5 in different types of glioma across the CGGA and TCGA databases (one‐way ANOVA). (B), (D) ROC curves for NSUN5 in the CGGA and TCGA. (E), (F) Kaplan‐Meier curves for NSUN5 in the CGGA and TCGA databases. (G), (H) Forest plots of multivariate Cox regression analyses in the CGGA and TCGA cohorts.

The transcriptomic subtypes of gliomas have been widely recognized, with classical and mesenchymal subtypes associated with poorer prognosis (Verhaak et al. 2010). We investigated the distribution of NSUN5 across glioma subtypes and found that NSUN5 was highly expressed in classical and mesenchymal subtypes in the CGGA database (Figure 4A). The specificity of this finding was supported by an ROC curve with an AUC of 0.806 (Figure 4B). The TCGA database further validated the robustness of our findings, with an AUC of 0.783 in the ROC analysis (Figure 4C,D). Finally, we performed Kaplan–Meier survival and Cox proportional hazards analyses based on both CGGA and TCGA datasets to further evaluate the prognostic value of NSUN5 in glioma patients. In the CGGA dataset, patients with high NSUN5 expression showed significantly shorter OS (median survival: 484 days) compared to those with low expression (median survival: 1126 days) (Figure 4E). Validation using the TCGA database further confirmed the prognostic value of NSUN5 (Figure 4F). In Cox regression analysis, NSUN5 expression was identified as an independent prognostic factor, showing no significant correlation with other established prognostic variables such as WHO grade, age at diagnosis, IDH mutation status, and 1p/19q codeletion (Figure 4G,H).

### 3.4 Immune Infiltration Analysis

**FIGURE 5:**
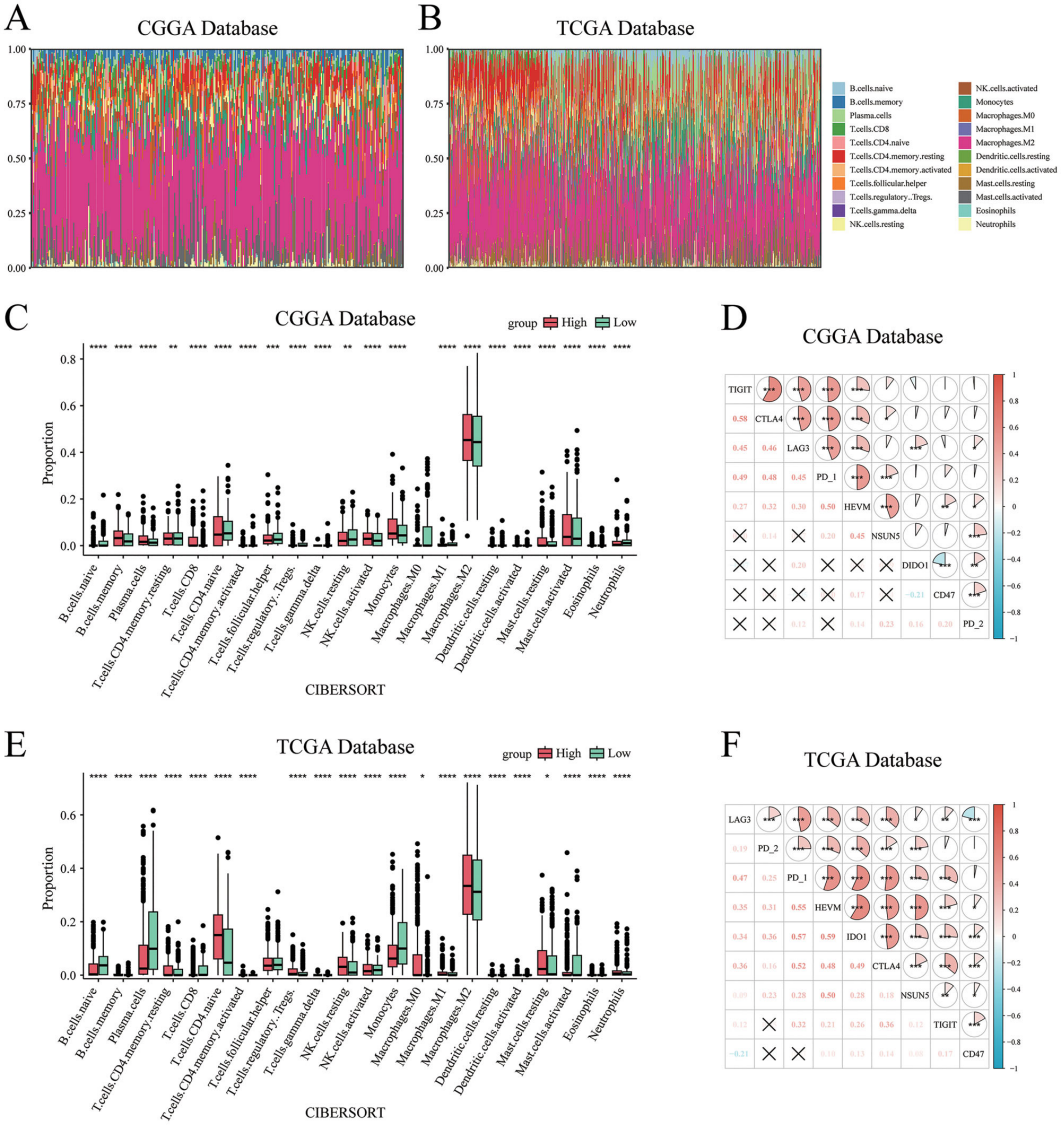
Impact of NSUN5 expression on immune infiltration in glioma. (A), (B) Stacked bar plots showing the abundance of immune cell infiltration in the CGGA and TCGA datasets. (C), (E) Correlation analysis between high and low NSUN5 expression groups and 22 immune cell types identified by the CIBERSORT algorithm in the CGGA and TCGA datasets. (D), (F) Pearson correlation matrices showing the association between NSUN5 expression and immune checkpoint molecules in the CGGA and TCGA datasets.

To investigate the relationship between NSUN5 expression and the tumor immune microenvironment, we used the CIBERSORT algorithm to estimate the relative abundance of 22 immune cell types (Figure 5A,B). Significant differences in immune cell infiltration were observed between the NSUN5 high‐ and low‐expression groups in both the CGGA and TCGA datasets, particularly with a marked increase in M2 macrophage infiltration in the high‐expression group (Figure 5C,E). In addition, we analyzed the correlation between NSUN5 expression and several inhibitory immune checkpoints, including TIGIT, CTLA4, LAG3, DIDO1, CD47, PD‐1, PD‐2, and HVEM. The analysis revealed that NSUN5 expression was positively correlated with the majority of these inhibitory immune checkpoints (Figure 5D,F).

### 3.5 Drug Screening and Target Prediction Analysis

**FIGURE 6:**
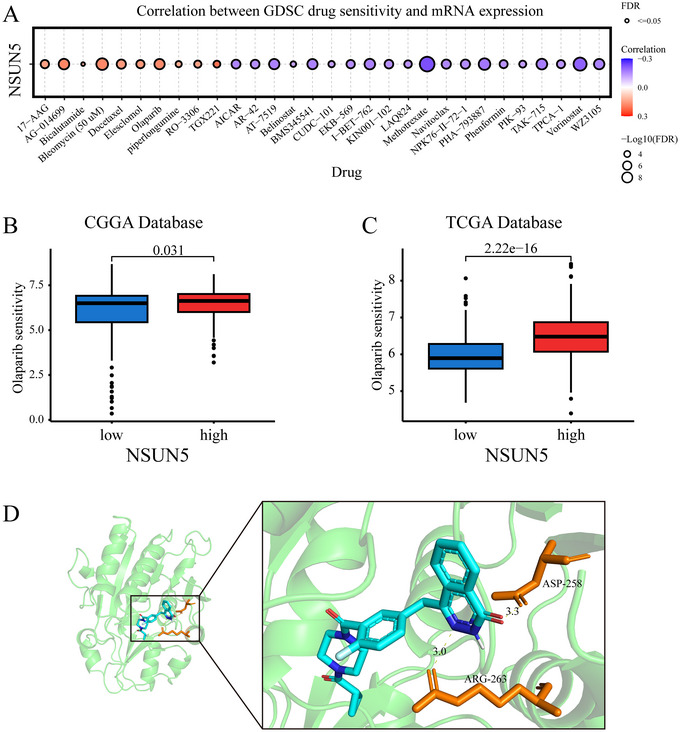
Drug screening and target prediction analysis of NSUN5. (A) Sensitivity of the top 30 drugs most significantly associated with NSUN5 in the GDSC database. (B), (C) Correlation analysis between NSUN5 expression and Olaparib sensitivity in the CGGA and TCGA datasets. (D) Molecular docking results between NSUN5 protein and Olaparib.

First, based on the GDSC database, we identified the top 30 drugs most significantly associated with NSUN5 sensitivity in solid tumors (Figure 6A). Subsequently, drug response analyses from the CGGA and TCGA databases revealed a significant positive correlation between NSUN5 expression and sensitivity to Olaparib (Figure 6B,C). To further investigate a potential direct interaction between NSUN5 and Olaparib, we performed molecular docking analysis. The results showed that the NSUN5 protein can directly interact with the target of Olaparib, exhibiting a stable binding affinity (−8.8 kcal/mol) (Figure 6D).

### 3.6 Functional Enrichment Analysis of NSUN5

**FIGURE 7:**
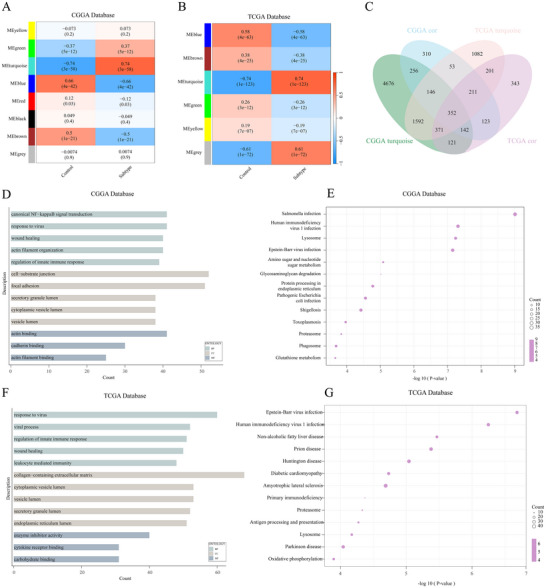
GO and KEGG enrichment analysis. (A), (B) WGCNA analysis in the CGGA and TCGA databases. (C) Venn diagram of the overlap between WGCNA‐derived gene modules and NSUN5‐related genes in the CGGA and TCGA datasets. (D), (E) GO and KEGG analysis in the CGGA database. (F), (G) GO and KEGG analysis in the TCGA database.

We conducted WGCNA analysis using the transcriptomic subtypes of gliomas. We identified the most relevant gene module, turquoise (Figure 7A,B), and selected the gene sets most strongly associated with NSUN5 for GO and KEGG analysis (Figure 7C). In the CGGA database, the most enriched biological processes included canonical NF‐kappaB signal transduction, response to viruses, wound healing, actin filament organization, and regulation of the innate immune response; the associated cellular components were vesicle lumen, secretory granule lumen, and endoplasmic reticulum lumen; and the related molecular function was actin binding (Figure 7D). Similar enrichment results were observed in the TCGA database (Figure 7F). The KEGG results from the CGGA and TCGA databases revealed that NSUN5 is closely associated with Epstein‐Barr virus infection, human immunodeficiency virus one infection, lysosome function, and proteasome (Figure 7E,G).

### 3.7 Establishing a Prognostic Model Associated With NSUN5 Using Mime

**FIGURE 8:**
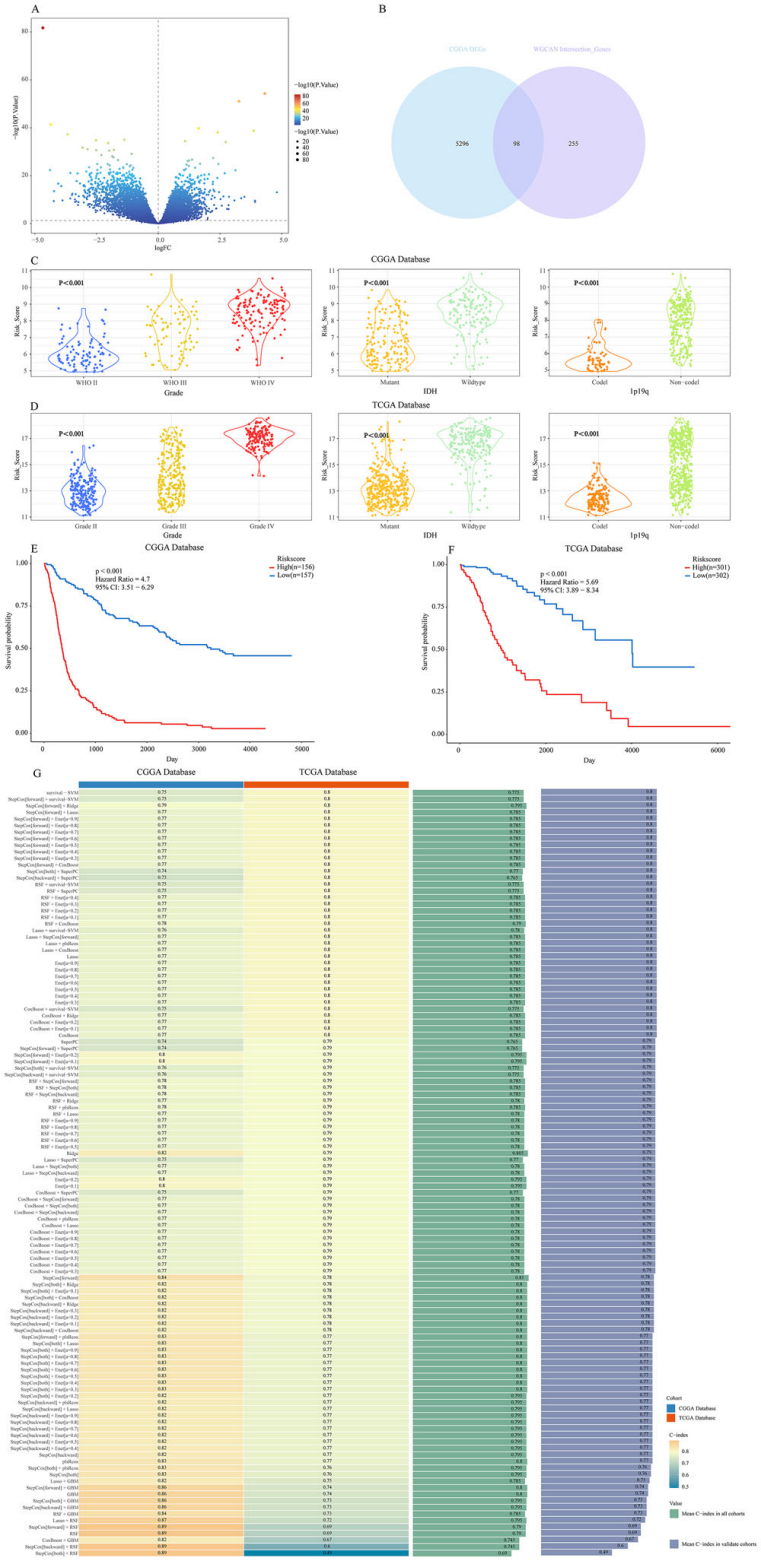
Construction of an NSUN5‐related prognostic model using the Mime framework. (A) Volcano plot of differentially expressed genes in the CGGA database. (B) Venn diagram of differentially expressed genes and WGCNA‐derived genes in the CGGA dataset. (C), (D) Relationship between risk scores and clinical pathology of glioma patients in the CGGA and TCGA databases. (E), (F) K‐M curves of risk scores in the CGGA and TCGA databases. (G) The c‐index of each model in the CGGA and TCGA databases is sorted by the average C‐index in the validation cohort.

A total of 5394 differentially expressed genes were identified in the CGGA dataset (Figure 8A). By integrating WGCNA results from both the CGGA and TCGA datasets, 352 genes were identified as being associated with NSUN5. The intersection of the differentially expressed genes and the NSUN5‐associated genes yielded 98 candidate genes (Figure 8B). We selected the model with the highest average C‐index, StepCox[forward] + Ridge (STRICOM), from among 117 machine‐learning models (Figure 8G). We calculated a risk score for each glioma patient using the Mime package. In the CGGA database, we observed that as the risk score increased, glioma patients exhibited more aggressive pathological features (Figure 8C). The median risk score calculated by STRICOM further stratified glioma patients into high‐risk and low‐risk groups, predicting their survival probability in each cohort. Patients with high‐risk scores had significantly worse outcomes (Figure 8E). These results were validated in the TCGA validation cohort (Figure 8D,F).

### 3.8 Efficacy Evaluation of the Optimal Model

**FIGURE 9:**
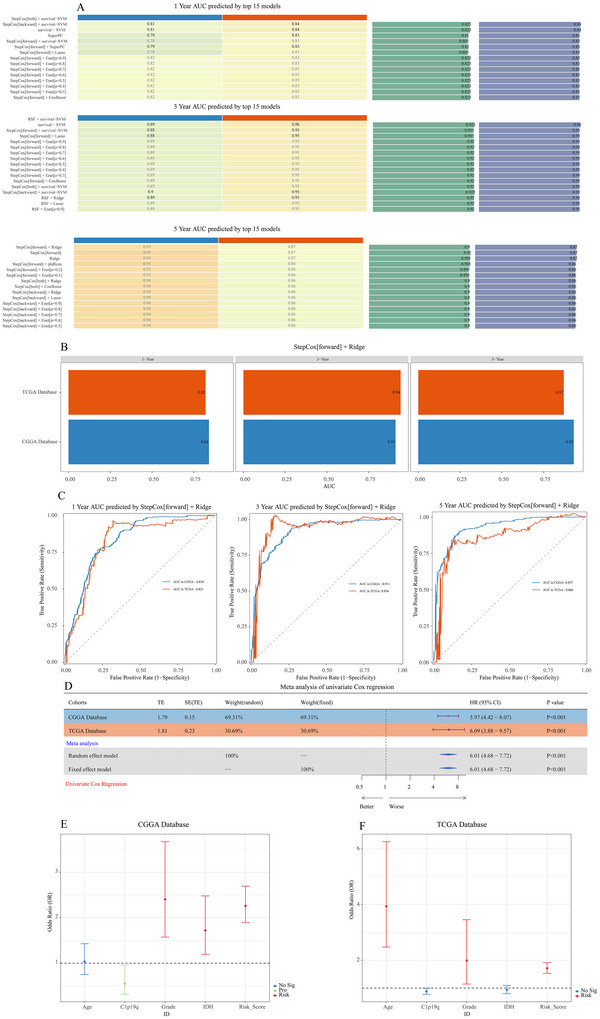
Performance of the prognostic model. (A) 1‐year, 3‐year, and 5‐year AUC for the top 15 models in the CGGA and TCGA databases, sorted by the average AUC in the validation cohort. (B) 1‐year, 3‐year, and 5‐year AUC for the STRICOM combined model in the CGGA and TCGA databases. (C) 1‐year, 3‐year, and 5‐year ROC curves in the CGGA and TCGA databases. (D) Meta‐analysis of univariate Cox results for the STRICOM combined model in the CGGA and TCGA databases. (E), (F) Forest plot of multivariate Cox results for the STRICOM combined model in the CGGA and TCGA databases.

As AUC is a commonly used metric for evaluating prognostic models, we used the Mime package to perform ROC curve analysis on STRICOM. The 1‐year, 3‐year, and 5‐year ROC curves for STRICOM, predicted in both the training cohort (CGGA) and the validation cohort (TCGA), demonstrated good predictive performance, with AUC > 0.8 (Figure 9B,C). Although the 1‐year and 3‐year AUC for STRICOM were not the highest, the 5‐year AUC ranked first in the validation cohort (Figure 9A). To assess the prognostic impact of STRICOM, we conducted a meta‐analysis of univariate Cox regression using the Mime package. The results indicated that the STRICOM‐calculated score is a significant risk factor for glioma (Figure 9D). After identifying several known molecular biomarkers for glioma, we conducted multivariate Cox regression analysis, which revealed that the STRICOM‐calculated score is an independent prognostic factor. The age at diagnosis, WHO grade, IDH mutation, and 1p/19q codeletion in glioma patients were not significantly associated with prognosis (Figure 9E,F). These results suggest that the optimal Mime model accurately predicts patient prognosis.

### 3.9 Comparison Between the Optimal Model and Other Models

**FIGURE 10:**
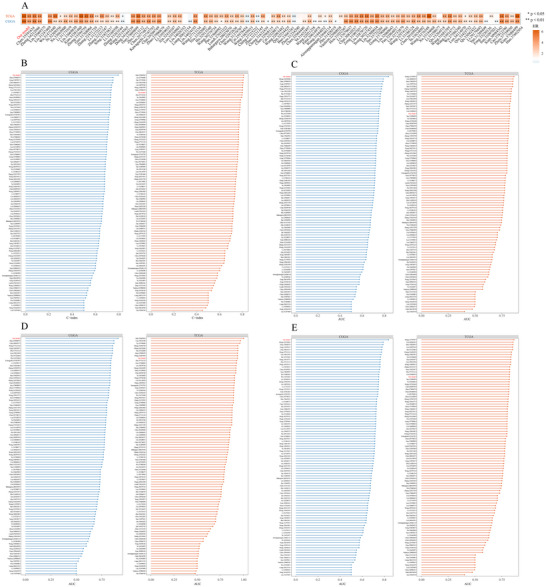
Comparison between the optimal model and other models. (A) Hazard ratios (HRs) of the STRICOM model compared with 95 previously published models. (B) Comparison of the C‐index between the STRICOM model and 95 previously published models. (C), (E) Comparison of 1‐, 3‐, and 5‐year AUC values between the STRICOM model and 95 previously published models.

Numerous machine learning‐based prognostic and predictive models have recently been applied to gliomas (Zhang et al. 2022). To comprehensively compare STRICOM with other published glioma models, we retrieved 95 such models from previous studies. In this study, we performed univariate Cox regression analyses on the CGGA and TCGA datasets to evaluate the association between the STRICOM model and patient prognosis. We found that, compared with other models, STRICOM was significantly associated with poor prognosis in glioma patients in both the CGGA and TCGA cohorts (Figure 10A). Moreover, the STRICOM model achieved among the highest rankings in terms of C‐index as well as 1‐, 3‐, and 5‐year AUC values across all models (Figure 10B–E). These results indicate that STRICOM has superior prognostic performance.

### 3.10 Immune Infiltration Analysis of the Optimal Model

**FIGURE 11:**
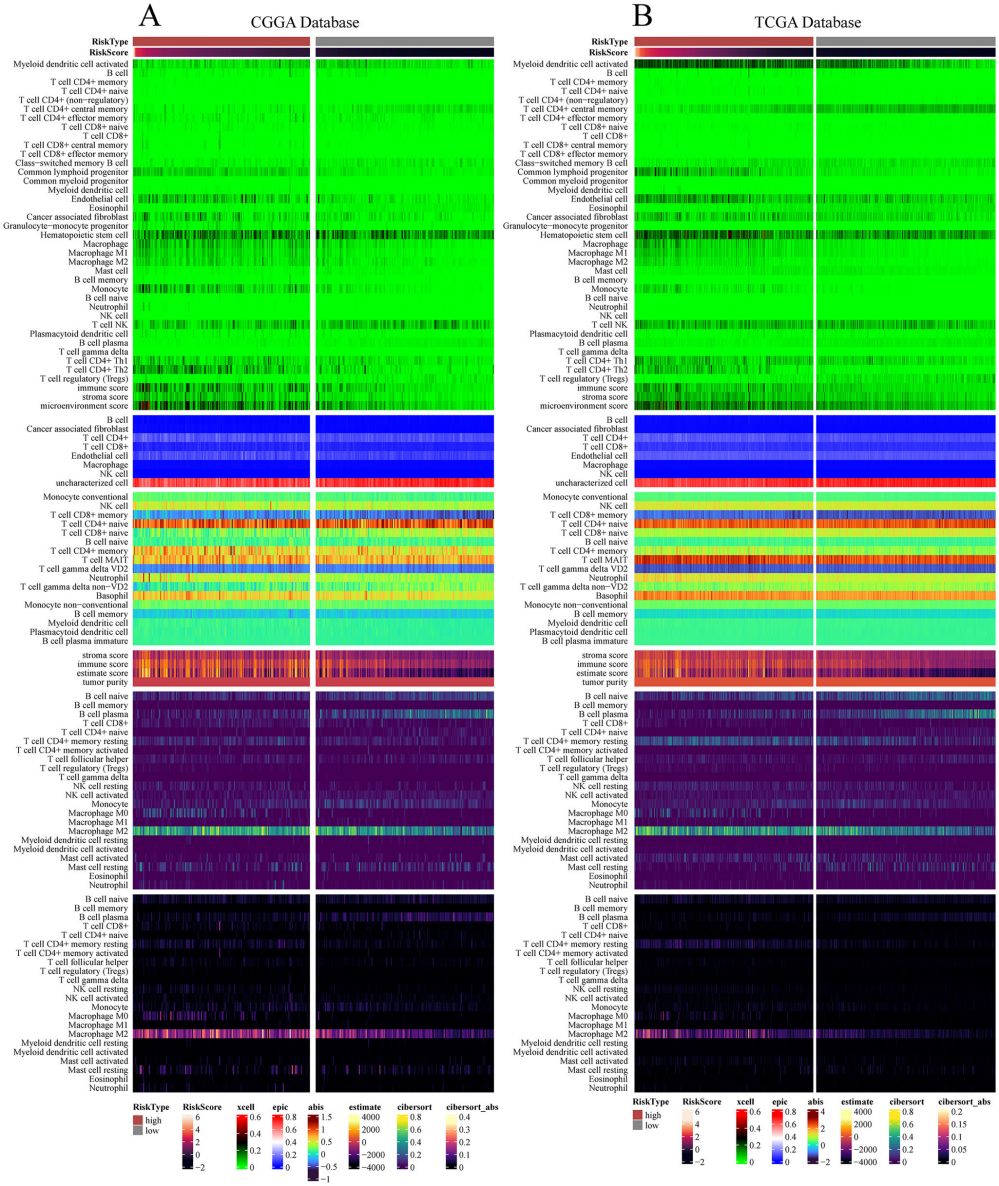
Correlation between risk score and immune or genome signatures. (A) Correlation between STRICOM model–derived risk scores and tumor microenvironment features estimated by various deconvolution methods in the CGGA dataset. Method IPS was from package IOBR, while other methods were from package immunedeconv. (B) Correlation between STRICOM‐combined model–derived risk scores and tumor microenvironment features estimated by various deconvolution methods in the TCGA dataset.

To facilitate downstream analysis following the construction of the prognostic model, Mime integrates immune infiltration and tumor microenvironment (TME) features from the R packages immunedeconv (Sturm et al. 2020, 2019) and IOBR (Zeng et al. 2021), allowing users to visualize the results quickly. Through TME analysis, we observed that, in the TCGA and CGGA cohorts, the high‐risk group exhibited higher immune infiltration scores compared to the low‐risk group in STRICOM. Furthermore, in the Cibersort analysis, the high‐risk group exhibited predominant expression of M2 macrophages (Figure 11A,B).

### 3.11 Construction of the Nomogram

**FIGURE 12:**
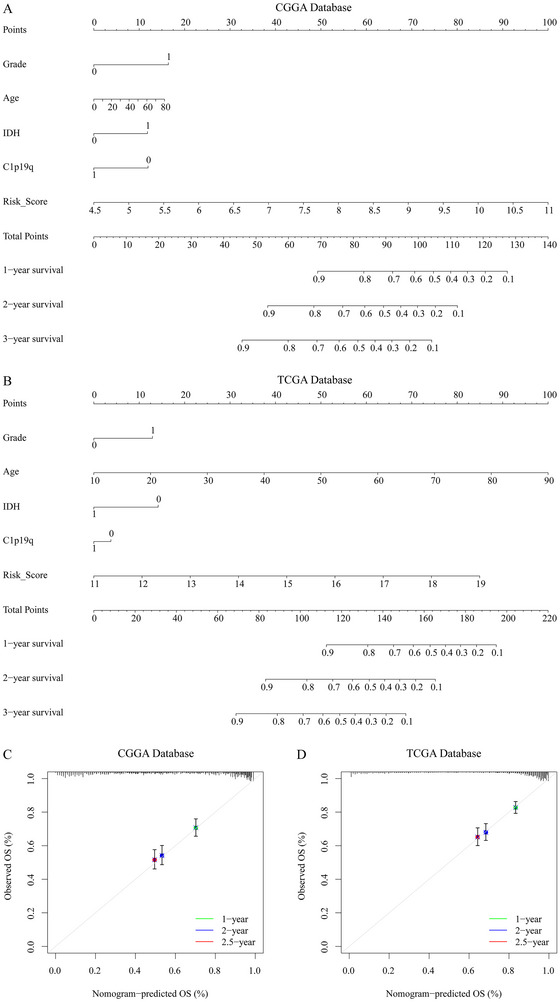
Construction and validation of the nomogram. (A), (B) Nomograms for predicting overall survival in glioma patients based on the CGGA and TCGA datasets. (C), (D) Calibration curves for evaluating the predictive performance of the nomograms in the CGGA and TCGA datasets.

We constructed a predictive model based on WHO grade, age, 1p/19q status, and risk score, which predicts the 1‐year, 2‐year, and 3‐year survival rates of glioma patients (Figure 12A–B), and the calibration curves show that the actual survival rates of glioma patients are consistent with the expected rates (Figure 12C,D). In conclusion, the nomogram demonstrates good predictive ability for glioma patients.

### 3.12 Cellular and Histological Experiments Validate the Expression of NSUN5

**FIGURE 13:**
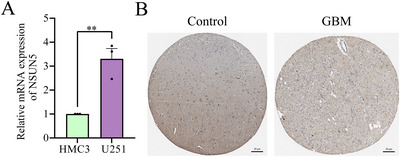
Tissue and cellular experiments related to NSUN5. (A) qPCR analysis of NSUN5 expression in glioma. (B) immunohistochemistry staining of NSUN5 in glioma tissues.

To confirm our previous speculations, we conducted relevant organizational and cellular experiments. Utilizing RT‐Qpcr and, we investigated the expression of NSUN5 in glioma tissues, with results consistent with RNA sequencing data, indicating higher expression of NSUN5 in glioma (Figure 13A). The HPA online database also revealed that NSUN5 expression was notably upregulated in glioma patients (Figure 13B).

## 4 Discussion

Glioma is the most common and aggressive malignant tumor of the central nervous system, particularly GBM, which has an inferior prognosis. Despite treatments such as surgical resection, radiotherapy, and chemotherapy, the median survival time of patients remains short (Stupp et al. 2005). Advances in molecular biology have identified several potential prognostic markers for glioma, offering new avenues for targeted therapy (Colman 2020). Therefore, identifying suitable molecular markers for glioma holds promise for developing new therapeutic strategies.

5‐Methylcytosine (m5C) modification is one of the most abundant modifications in eukaryotic cells (Squires et al. 2012). This modification mainly occurs in rRNA, tRNA, and mRNA, potentially affecting RNA stability, translation efficiency, and protein expression (Chellamuthu and Gray 2020). In eukaryotic cells, RNA m5C modification is typically catalyzed by the NOL1/NOP2/SUN domain (NSUN) family of enzymes and the DNA methyltransferase homolog DNMT2 (Hu et al. 2021). Members of the NSUN family play significant roles in tumor development. NSUN1/NOP2 promotes the migration and invasion of lung cancer cells in vitro, thereby enhancing tumor growth and progression in vivo (Yang et al. 2024). NSUN2 is mainly responsible for the m5C methylation of tRNA and mRNA. In gastric cancer, it promotes tumor cell proliferation, migration, and invasion by upregulating FOXC2, and it is associated with poor prognosis in patients (Yan et al. 2021). It is also overexpressed in liver cancer, promoting cancer cell proliferation, migration, invasion, and angiogenesis by upregulating FZR1 and H19 while inhibiting apoptosis (Sun et al. 2020; Wang et al. 2014). NSUN3 primarily methylates mitochondrial tRNA, maintaining mitochondrial function. The overexpression of NSUN3 enhances the proliferation and invasion of hypopharyngeal squamous cell carcinoma by upregulating TEAD1 (Chen et al. 2021). NSUN4 also plays a role in liver and lung squamous cell carcinoma (He et al. 2020; Pan et al. 2021). NSUN5 shows abnormal expression in various cancers, including gastric cancer, clear cell renal carcinoma, colorectal cancer, liver cancer, and head and neck squamous cell carcinoma, playing a key role in tumor initiation and progression.

In recent years, treatment strategies for low‐grade gliomas have progressively evolved from conventional surgery and radiotherapy to encompass immunotherapies (e.g., Zotiraciclib, Lerapolturev), targeted agents (e.g., Entrectinib, ONC201/Paxalisib combination), and vaccines (e.g., synthetic RNA adjuvants), advancing the individualized and precision‐based management of gliomas (Lucke‐Wold et al. 2024). Advances in imaging modalities have provided critical tools for precision medicine. Saraswathy et al. (2009) demonstrated that preoperative multimodal MRI—including T2 hyperintense non‐enhancing regions, diffusion‐weighted imaging, and proton magnetic resonance spectroscopic imaging—effectively predicts survival in GBM patients, serving as imaging biomarkers for prognosis and informing individualized therapeutic decisions. Furthermore, Hey et al. (2023) revealed that glioma‐associated pathogenic mechanisms may significantly alter the expression and structure of ligand‐gated ion channels, thereby promoting tumor progression and heterogeneity, supporting their potential as diagnostic and therapeutic targets. However, Ganau et al. (2015) highlighted that despite ongoing therapeutic advances for both low‐grade and high‐grade gliomas, challenges persist, including slow translation of basic research findings and a paucity of high‐quality multicenter randomized clinical trials, underscoring the necessity for individualized, patient‐centered management strategies. Therefore, identifying molecular biomarkers with dual diagnostic and therapeutic value is essential for optimizing personalized treatment. This study focuses on the RNA methyltransferase NSUN5, which not only possesses molecular diagnostic potential but may also contribute to glioma progression through modulation of the TME, thereby offering a novel target for precision therapy.

In glioma tissues, low expression levels of NSUN5 are more pronounced in low‐grade gliomas. They are positively correlated with more prolonged OS across all grades of glioma (Galardi et al. 2020), which corroborates our findings. Additionally, we found that NSUN5 is prone to genetic mutations in glioma patients, and those with mutations exhibit significantly reduced OS and disease‐specific survival. Research by Janin et al. demonstrated that the loss of NSUN5 inhibits the methylation of the C3782 site on 28S rRNA, leading to a reduction in overall protein synthesis. This forces cells to adopt adaptive translation programs under stress, increasing translation efficiency to overcome oxidative stress encountered during differentiation, thereby extending the OS of glioma patients (Janin et al. 2019; Zhou et al. 2023). However, NSUN5 methylation‐mediated overexpression of NQO1 presents a therapeutic opportunity in these gliomas, as cells exhibit increased sensitivity to ROS‐induced death when treated with NQO1 bioactive molecules (Lei et al. 2020; Wang et al. 2023). Thus, one future direction for glioma treatment is to inhibit the growth and development of glioma cells by targeting therapeutic targets. Drug therapy is currently one of the primary clinical methods for combating tumors. Research shows that Olaparib can inhibit mitochondrial complex I, thereby affecting DNA repair and the repair of respiratory damage in glioma cells. Furthermore, Olaparib can be used in combination with other drugs to enhance its antitumor effects (Saad et al. 2023; Wang et al. 2024; Zampieri et al. 2021). Our results indicate that the therapeutic efficacy of Olaparib is closely associated with the expression level of NSUN5. Therefore, NSUN5 may be a key factor influencing glioma invasiveness and prognosis and could serve as a potential therapeutic target.

The limited benefit of immunotherapy for glioma patients is due to the unique immunosuppressive microenvironment of GBM, which inhibits both systemic and local immune responses, leading to lower levels of lymphocyte infiltration (Waldman et al. 2020). Research by Zhang et al. (2021) indicates that tumor‐associated macrophages (TAMs) are abundant in the TME, and M2‐type macrophages significantly promote glioma progression. Pan and Strahle (2025)demonstrated that cerebrospinal fluid plays a critical pathological role in glioma progression by interacting with interstitial fluid to promote TME formation and tumor cell migration. Watson et al. treated glioma with a monoclonal antibody targeting CSF‐1R to specifically inhibit the activity and proliferation of macrophages, thereby reducing tumor‐associated immunosuppression. Fibrotic response to anti‐CSF‐1R therapy potentiates GBM recurrence. Snacel‐Fazy et al. (2024) used the SMAC mimetic GDC‐0152 SMg to promote pro‐apoptotic and antitumor functions of macrophages by inducing caspase‐3‐mediated inflammatory cleavage. Therefore, we can promote the body's antitumor response by enhancing M1‐type macrophages or inhibiting M2‐type macrophages. Our results show a close correlation between NSUN5 and immune cells. Using machine learning core algorithms, we found a significant positive correlation between the infiltration of M2‐type macrophages and high‐risk patient groups. NSUN5 promotes the degradation of CTNNB1 caRNA by depositing m^5^C modifications, thereby downregulating β‐catenin expression. This indirectly suppresses CD47 expression and enhances macrophage‐mediated phagocytosis of glioma cells, highlighting NSUN5's potential role as an upstream regulator of the immune checkpoint CD47 (Wu et al. 2024). Furthermore, Mortezaee et al. (2024) reported that hyperactivation of the WNT/β‐catenin pathway inhibits dendritic cell (DC) recruitment, impairs CD8^+^ T cell infiltration, increases the proportion of regulatory T cells (Tregs), and upregulates PD‐L1 and PD‐1 expression. Our analysis revealed a significant correlation between NSUN5 expression and multiple immune checkpoint molecules, suggesting a potential role for NSUN5 in modulating tumor immune evasion. Therefore, targeting the regulation of NSUN5 expression in glioma patients to alter the tumor immune microenvironment and promote the body's immune response to tumor cells is expected to become a new strategy for glioma treatment.

Although NSUN5 has excellent potential as a prognostic marker in gliomas, it still faces several challenges. First, there are significant differences in the molecular characteristics of different patients, and how to precisely distinguish the Relationship between NSUN5 expression levels and other prognostic factors requires further investigation. Secondly, although studies have preliminarily uncovered the function of NSUN5 in tumors, its specific molecular mechanisms, especially its role in gliomas, still need to be further elucidated. Understanding these mechanisms will lay the theoretical foundation for developing NSUN5‐targeted therapeutic strategies.

## 5 Conclusion

NSUN5, as an RNA methyltransferase, plays a key role in the onset and progression of gliomas, and its abnormal expression is closely associated with patient survival outcomes. As a potential prognostic marker and therapeutic target, NSUN5 may provide critical insights for personalized glioma treatment and advance the precision of clinical interventions. Therefore, in‐depth research into the diagnostic, prognostic evaluation, and therapeutic roles of NSUN5 in gliomas could bring new hope for improving patient survival rates.

## Author Contributions

**Ye Wenhao**: formal analysis, validation, visualization, writing – original draft, methodology. **Wu Huan**: validation, writing – original draft, methodology. **Zou Xiaoyun**: data curation, writing – original draft, validation. **Yang Yuanyuan**: data curation, writing – original draft, validation. **Bi Junlei**: data curation, writing – original draft, validation. **Liu Changqing**: data curation, writing – original draft. **Zhao Mengyi**: data curation, writing – original draft. **Zhang Yuyuan**: data curation, writing – original draft. **Lu Jin**: data curation, writing – original draft. **Wen Hebao**: funding acquisition, project administration, supervision, writing – review and editing. **Ma Caiyun**: project administration, resources, supervision, writing – review and editing.

## Funding

This work was supported by the National Natural Science Foundation of China (82371382), Natural Science Foundation of the Higher Education Institutions of Anhui Province (2024AH051296), Quality Engineering Project of Higher Education Institutions of Anhui Province (2023jyxm0653), Longhu Talent Project of Bengbu Medical University (LH250204001), Anhui Provincial Young Backbone Teachers’ Domestic Visiting and Research Training Program (JNFX2025034), and the Experimental Teaching and Teaching Laboratory Quality Engineering Project of Bengbu Medical University(2024syyj05).

## Ethics Statement

The authors have nothing to report.

## Conflicts of Interest

The authors declare no conflicts of interest.

## Supporting information



Supplementary Table: brb371211‐sup‐0001‐Table S1.xlsx

Supplementary Table: brb371211‐sup‐0002‐Table S2.xlsx

Supplementary Table: brb371211‐sup‐0003‐Table S3.xlsx

Supplementary Table: brb371211‐sup‐0004‐Table S4.xlsx

## Data Availability

Publicly available datasets were analyzed in this study. This data can be found here: TCGA (https://cancergenome.nih.gov), and CGGA (http://www.cgga.org.cn/index.jsp).
